# Camera-based automated monitoring of flying insects (Camfi). I. Field and computational methods

**DOI:** 10.3389/finsc.2023.1240400

**Published:** 2023-09-13

**Authors:** Jesse Rudolf Amenuvegbe Wallace, Therese Maria Joanna Reber, David Dreyer, Brendan Beaton, Jochen Zeil, Eric Warrant

**Affiliations:** ^1^ Research School of Biology, The Australian National University, Canberra, ACT, Australia; ^2^ National Collections & Marine Infrastructure, CSIRO, Parkville, VIC, Australia; ^3^ Lund Vision Group, Department of Biology, Lund University, Lund, Sweden

**Keywords:** Camfi, population monitoring, flight behaviour, insect conservation, insect ecology, remote sensing, computer vision, image analysis

## Abstract

The ability to measure flying insect activity and abundance is important for ecologists, conservationists and agronomists alike. However, existing methods are laborious and produce data with low temporal resolution (e.g. trapping and direct observation), or are expensive, technically complex, and require vehicle access to field sites (e.g. radar and lidar entomology). We propose a method called “Camfi” for long-term non-invasive population monitoring and high-throughput behavioural observation of low-flying insects using images and videos obtained from wildlife cameras, which are inexpensive and simple to operate. To facilitate very large monitoring programs, we have developed and implemented a tool for automatic detection and annotation of flying insect targets in still images or video clips based on the popular Mask R-CNN framework. This tool can be trained to detect and annotate insects in a few hours, taking advantage of transfer learning. Our method will prove invaluable for ongoing efforts to understand the behaviour and ecology of declining insect populations and could also be applied to agronomy. The method is particularly suited to studies of low-flying insects in remote areas, and is suitable for very large-scale monitoring programs, or programs with relatively low budgets.

## 1 Introduction

The ability to measure flying insect activity and abundance is important for ecologists, conservationists and agronomists alike. Traditionally, this is done using tedious and invasive methods including nets (e.g. 1), window traps (e.g. 2), light traps (e.g. 3, 4), and pheromone traps (e.g. 5, 6), with the latter being favoured by agronomists for its specificity. The WWII development of radar led to the introduction of radar ornithology (7, 8) and ultimately radar entomology (9, 10), which facilitated non-invasive remote sensing of insects flying up to a couple of kilometres above the ground, and became extremely important for understanding the scale and dynamics of insect migration (11). More recently, entomological lidar has been introduced, which benefits from a number of advantages over radar, in particular the ability to measure insects flying close to the ground, without suffering from ground clutter (12, 13). However, both entomological radar and entomological lidar systems are relatively large (requiring vehicle access to study sites), bespoke, expensive, and require expertise to operate, reducing their utility and accessibility to field biologists.

We propose a method for long-term population monitoring and behavioural observation of low-flying wild insects using wildlife cameras. The proposed method, described herein, combines simple and inexpensive field techniques with an advanced, open-source, and highly automated computational processing workflow based on Mask R-CNN (14). The method therefore lends itself to large-scale studies and can generate substantial volumes of behavioural and abundance data, allowing for the detection of subtle interactions between external factors, and insect abundance and behaviour.

The method permits study designs in which cameras are deployed at fixed points—potentially over long durations—which are used to detect insects flying past the camera. It is therefore best suited to studies which can make use of detection count and flight telemetry data, but is not suitable for analysing inter-individual interactions or individual trajectories over a larger area. Depending on the research question, the method can make use of either still images or video clips. The former enables long-term population monitoring in remote areas with only occasional visits by the field worker and produces (relatively) compact datasets which are amenable to manual or automatic image-annotation, while the latter enables rapid measurement of oriented flight behaviour from large numbers of insects over short periods of time. Under certain circumstances, the method also facilitates crude measurements of wingbeat frequency, allowing the researcher to exclude non-target species with very different wingbeat frequencies from their analyses.

In this paper, we describe our new method, including algorithms for automatic annotation of images of flying insects and tracking multiple insects in video clips. We also present a command-line program and python library, called Camfi, which implements the described procedures and characterises the performance of the automated annotation algorithm. In the proceeding paper in this journal, we demonstrate the utility of the new method by measuring activity levels and flight behaviour of migratory Bogong moths (*Agrotis infusa*) over two summers in the Australian Alps (15). The Bogong moth is an important source of energy and nutrients in the fragile Australian alpine ecosystem (16), and is a model species for studying directed nocturnal insect migration and navigation (17–19). A dramatic drop in the population of Bogong moths has been observed in recent years (20, 21), adding it to the growing list of known invertebrate species whose populations are declining (22). The present method will prove invaluable for ongoing efforts to understand the behaviour and ecology of the Bogong moth, and to monitor the population of this iconic species. The new method allows for straight-forward training on new datasets and other flying insect species, giving it promising applications within insect conservation and agronomy beyond the Bogong moth study system to which it has currently been applied.

## 2 Methods

Where indicated, the methods described below have been automated in our freely available software and python library, called Camfi. A full practical step-by-step guide for using these methods along with complete documentation of the latest version of the code is provided at https://camfi.readthedocs.io/.

### 2.1 Data collection

**Figure 1:**
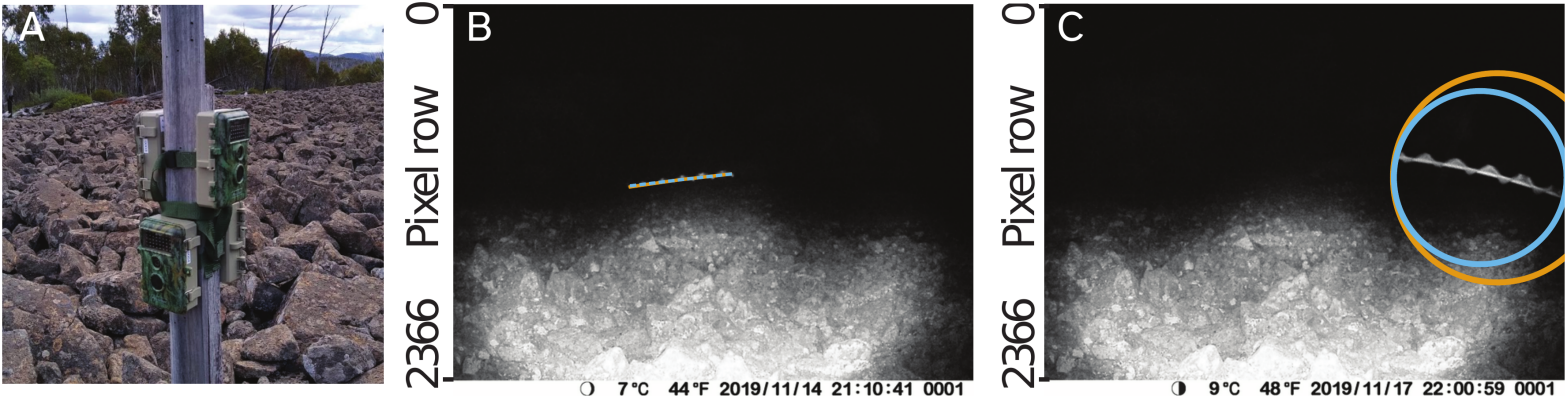
Example images showing data collection procedures used in this study. **(A)**. Wildlife cameras (BlazeVideo, model SL112) were set to capture still photos and/or videos on timers and were deployed at the study sites known for their abundance of target flying insects. Typically, cameras were placed facing the sky, but could also be placed on an elevated mount, such as a post (pictured). **(B)**. Motion blurs of moths captured by the cameras were marked with a polyline annotation. Manual annotation made in VIA (23) is shown in orange, and the annotation made by our automated procedure is shown in blue (although since both annotations are very similar, they overlap and only the blue annotation is visible). **(C)**. Circular or point annotations were used for images of moths whose motion blurs were not fully contained within the frame of the camera, or where the length of the motion blur was too short to see the moth’s wingbeat (latter case not shown). Manual annotation made in VIA (23) is shown in orange, and the annotation made by our automated procedure is shown in blue.

Images and video clips are collected in the field using wildlife cameras equipped with an infra-red LED flash and a “time-lapse mode” (we used BlazeVideo, model SL112, although most wildlife cameras or other infra-red cameras may be suitable). The cameras are positioned pointing towards the sky or other plain background (see sample placement in **Figure 1**), and set to take photos or short (e.g. 5 s at 30 frames per second, 1080p) video clips at regular intervals during the night (the PIR motion sensors on wildlife cameras generally do not trigger captures for invertebrates, as they depend on body heat, which limits them to detecting endotherms). The cameras are deployed in locations known for an abundance of flying insects of a known target species. When deployed in areas with a mixture of species, light traps can be deployed nearby to characterise the composition of the insect population in the area.

To train the automatic annotation model described below, we collected images from various locations known to be abundant in Bogong moths in the alpine areas of south-eastern Australia. These data are presented in further detail by Wallace et al. (15).

### 2.2 Image and video annotation

Our method permits three approaches to annotating the images and/or videos captured by the cameras in the field: 1. Manual annotation of still images using VIA (version 2; 23), 2. Automatic annotation of still images using Camfi, with optional validation or editing using VIA, and 3. automatic annotation of video clips using Camfi. When the target insects are flying sufficiently fast and the exposure time of the cameras is sufficiently long (which is typically the case when capturing photos or videos during the night), the insects appear as bright streaks on a dark background, due to motion-blur (**Figures 1B, C**). In annotation approaches 1 and 2 (and approach 3 for each individual video frame), the path of the motion-blur is annotated using a polyline for motion-blurs which are fully visible within the frame of the camera, or an enclosing circle when the motion-blur is partially occluded or out-of-frame. The geometries of the polyline annotations are later used by the wingbeat frequency measurement procedure (defined in **Supplementary Material S2**) and by the procedure for tracking multiple insects between video frames, described below.

#### 2.2.1 Approach 1: manual annotation of still images

Images are manually annotated for flying moths using VIA (23). A step-by-step guide to performing the annotations is provided in Camfi’s documentation (https://camfi.readthedocs.io/). Examples of polyline and circle annotations are displayed in **Figures 1B, C** (orange annotations).

We manually annotated 42420 images for flying insects. We reserved 250 images which contained annotations as a test set, and the rest were used for training the automatic annotation model.

#### 2.2.2 Approach 2: automated annotation of still images

Although the process of manually annotating the images is simple to undertake, it is also time-consuming, particularly for large volumes of images. For large-scale studies, it may be desirable to use automated annotation, either by itself or in conjunction with manual annotation. To that end, we have developed an automatic annotation tool, which is included with Camfi, and used by running `camfi annotate` from the command-line. The automatic annotation relies on Mask R-CNN (14), a state-of-the-art deep learning framework for object instance segmentation. The tool operates on VIA project files, allowing it to serve as a drop-in replacement for manual annotation. The tool also allows the annotations it generates to be loaded into VIA and manually edited if required. Examples of the annotations made by the automated procedure are displayed in **Figures 1B, C** (blue annotations).

A full description of the automated annotation procedure, including training, inference, and validation, is provided in the **Supplementary Material S1**. In summary, the procedure for model training uses the manual polyline annotations to create segmentation masks and bounding boxes which are used together with the images from which those annotations come from as training data for the Mask R-CNN model. Automated annotation uses the outputs of Mask R-CNN model inference (which are again segmentation masks and bounding boxes) to infer the geometries of polyline or circle annotations.

It should be noted that the automated annotation method uses polynomial regression to infer polyline annotations from the segmentation masks produced by Mask R-CNN (the order of the polynomial can be configured at run time). This is predicated on the target insects’ flight trajectories (within the course of a single exposure, e.g. 1/9 s) being able to be modelled with simple curves. In our experience, 2^nd^ order polynomials are sufficient, however a higher order may be required for insects which fly very tortuous paths.

We have pre-trained the annotation model on the manual annotations we made using approach 1 (above). Largely speaking, these annotations are of Bogong moths (a noctuid with ca. 5 cm wingspan). We expect the automated annotation to therefore work well for moths which are of a similar size and have a similar appearance while in flight. To simplify training of the model to target other species, we have implemented a tool which automates the training process, and this is described in the **Supplementary Material S1**. This tool is packaged with Camfi and is used by running `camfi train` from the command-line.

#### 2.2.3 Approach 3: automated video annotation

An advantage of Camfi is its flexibility regarding the temporal resolution of data collection. Depending on the research question, cameras can be set to capture an image at relatively long intervals, on the order of minutes, or they can be set to capture images at a very high rate, which in the case of video clips is on the order of hundredths of a second (typically 25-60 frames per second). However, when analysing Camfi data which have been obtained from high-rate captures (namely, videos), individuals will be detected multiple times, since each moth will be seen in each of many consecutive video frames as they pass by the camera. This results in detection counts being inflated by insects which have lower angular velocities relative to others from the perspective of the camera, and therefore spend more time in-frame. Therefore, to facilitate the use of videos by Camfi, we need to be able to track observations of individuals in a sequence of video frames, so we can count each individual only once.

In the following sections, we introduce an extension to Camfi which enables analysis of video data. This includes proper handling of video files, as well as tracking of individuals through consecutive frames. In addition to ensuring individual insects are only counted once per traversal of the camera’s field of view, the new method allows for measurement of the direction of displacement of insects as they travel through the air.

### 2.3 Multiple object tracking

Multiple object tracking is a challenging problem which arises in many computer vision applications, and which has been approached in a variety of different ways (reviewed by 24).

A common approach to multiple object tracking is “detection-based tracking” (also known as “tracking-by-detection”), in which objects are detected in each frame independently, and then linked together using one of a number of possible algorithms. Typically, this requires the use of a model of the motion of the objects to be tracked, along with a method which uses the model of motion to optimise the assignment of detections to new or existing trajectories. In many approaches, the modelled motion of tracked objects is inferred by combining information about the position of the objects in multiple frames. An obvious challenge arises here because the model of motion requires reliable identity information of objects detected in multiple frames, whereas the identity of the objects usually must be inferred from their motion (including their position). This circular dependency—between the inference of object identity and the model of motion—can be dealt with in a number of ways, including probabilistic inference via a Kalman filter (25) or a particle filter (e.g. 26), or through deterministic optimisation using a variety of graph-based methods.

Our approach to multiple object detection removes the requirement of an explicit model of motion entirely, by utilising two peculiar properties of the Camfi object detector. The first of these properties is that the Camfi detector obtains information about the motion of the flying insects it detects from the motion blurs the insects generate, which it stores in the form of a polyline annotation. Since this information is obtained from a single image, and therefore a single detection, it does not depend on the identity of the insect, solving the previously mentioned circular dependency problem. The second property is that the Camfi detector is robust to varying exposure times, owing to the fact it has been trained on images with a variety of exposure times. This in turn means that the detector is robust to the length of the insects’ motion blurs. Ultimately, these two properties, along with the fact that the insects appear as light objects on a dark background, mean that it is possible to use the Camfi detector to make a single detection of an individual insect traversing multiple consecutive frames. Thus, the trajectories can simply be formed using bipartite graph matching of overlapping polyline annotations, using only information provided by the detections themselves, using the method described below.

### 2.4 Automated flying insect tracking

**Figure 2:**
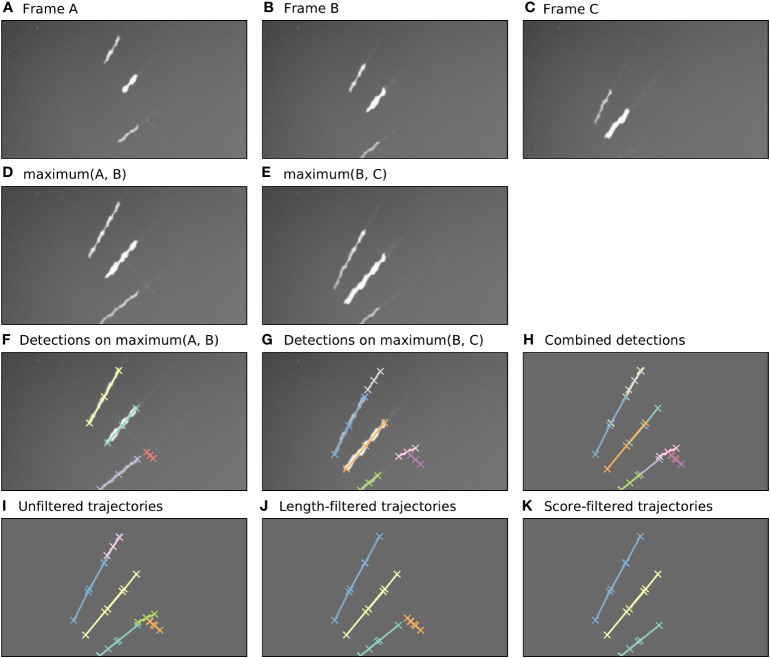
Automatic annotation is performed by Camfi on the maximum image of each pair of consecutive frames, allowing trajectories to be built from overlapping detections. Here, an example of this process is shown for three consecutive video frames. **(A–C)**. Three consecutive video frames containing multiple flying insects. **(D, E)**. The maximum image of each sequential pair of frames. **(F, G)**. Flying insects are detected in the two-frame maximum images using Camfi. **(H)**. Detections from **(F, G)** together on a plain background. **(I)**. Detections from sequential time-steps are combined into trajectories using bipartite graph matching on the degree of overlap between the detections. **(J)**. Trajectories containing fewer than three detections are removed. **(K)**. Finally, trajectories are filtered by mean detection score (trajectories with mean detection score lower than 0.8 are removed).

The algorithm for tracking flying insects in short video clips uses a detection-based tracking paradigm, relying heavily on the Camfi flying insect detector described above. An example of the sequence of steps taken by the tracking algorithm described in this section is illustrated in **Figure 2**. For brevity, the example shows the algorithm operating on three frames only, however the algorithm can operate on any number of frames, up to the memory constraints of the computer it is running on.

First, the video frames are prepared for flying insect detection. A batch of frames is loaded into memory (e.g. **Figures 2A–C**, although typically this would be a video clip). The maximum image of each sequential pair of frames is then calculated by taking the maximum (brightest) value for each pixel between the two frames (**Figures 2D, E**). This produces images with lengthened motion blurs of the in-frame flying insects, approximating the images which would be obtained if the exposure time of the camera were doubled. Importantly, the motion blurs of an individual insect in consecutive time-steps overlap each other in these maximum images.

Detection of flying insects is performed on the maximum images using the Camfi detector, producing candidate annotations of insect motion blurs to be included in trajectories (**Figures 2F, G**). The Camfi detector produces polyline annotations which follow the respective paths of the motion blurs of flying insects captured by the camera. Because the motion blurs of individual insects overlap in consecutive frames, so too do the annotations of those blurs (e.g. **Figure 2H**). This enables the construction of trajectories by linking overlapping sequential detections.

Detections in successive time-steps are linked by solving the linear sum assignment problem using the modified Jonker-Volgenant algorithm with no initialisation, as described by Crouse (27). In order to do this, a formal definition of the cost of linking detections is required. We call this cost the “matching distance”, which we denote by $d_{M}$ . Consider two polyline annotations $P_{a}$ and $P_{b}$ , which are sequences of line segments defined by the sequences of vertices ${\left(a_{i}\right)}_{i=0}^{n-1}$ and ${\left(b_{j}\right)}_{j=0}^{m-1}$ , respectively, where $a_{i},b_{i}\in {\mathbb{R}}^{2}.$ . We define $d_{M}\left(P_{a},P_{b}\right)$ as the second smallest element in $\left\{d\left(a_{0},P_{b}\right),\;d\left(a_{n-1},P_{b}\right),\;d\left(b_{0},\;P_{a}\right),\;d\left(b_{m-1},P_{a}\right)\right\}$ , where d(x,P) is the Euclidean distance from a point $x\in {\mathbb{R}}^{2}$ to the closest point in a polyline $P\subset {\mathbb{R}}^{2}$ . This definition of $d_{M}$ is efficient to compute, and allows us to discriminate between pairs of detections which come close to each other by chance (perhaps at very different angles) and pairs of detections which closely follow the same trajectory (i.e. roughly overlap each other).

After solving the assignment problem, a heuristic is applied to reduce spurious linking of detections into trajectories, where links with $d_{M}$ values above a specified threshold are removed. Trajectories are built across the entire batch of frames by iteratively applying the detection linking procedure for each consecutive pair of time-steps (**Figure 2I**). Trajectories containing fewer than three detections are removed (**Figure 2J**), as are trajectories with low mean detection scores (**Figure 2K**). The threshold for what is considered a low detection score can be set by the user (e.g. a value of 0.8 might be reasonable). When analyses relating to flight track directions are required, an additional filtering step can be applied to constrain analysis to detections inside a circular region of interest within the frame. This eliminates directional bias arising from the non-rotationally symmetrical rectangular shape of the video frames.

**Figure 3:**
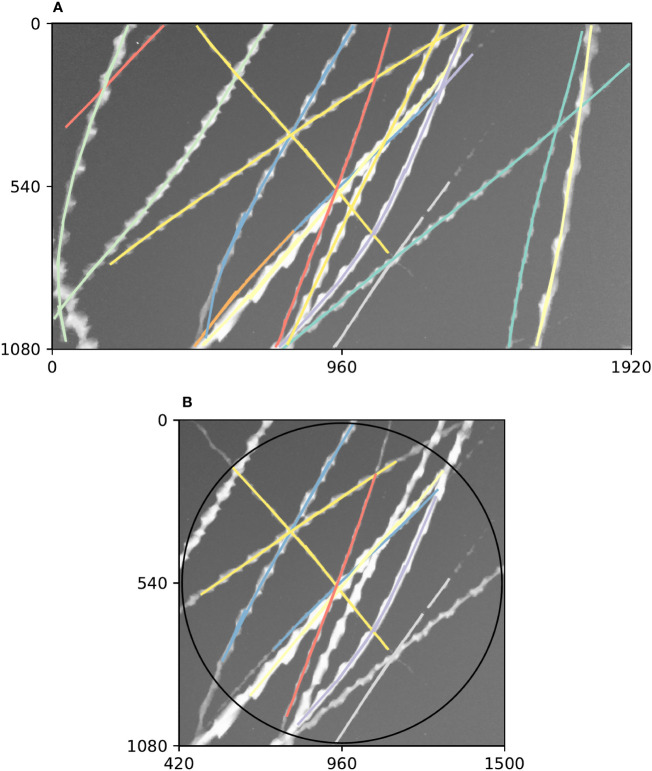
Example summary of trajectories followed by insects flying past a camera during a 5 s video clip. Axes on both plots show pixel row and column numbers. **(A)**. Maximum (brightest) value of each pixel across every frame in the clip with annotations overlaid. Visible bright streaks are made by the motion blurs of Bogong moths flying past the camera. The colour of an annotation indicates its membership in a unique trajectory, as predicted by our method. **(B)**. Annotations constrained to circular region of interest. Using only these trajectories eliminates directional bias resulting from the non-rotationally symmetrical rectangular shape of the frame. Black circle shows region of interest.

Diagnostic plots of tracking performance over an entire short video clip can be made by taking the maximum image of the entire video clip, and plotting the detected trajectories as a single image using a different colour for each trajectory (e.g. **Figure 3**). For example, we can see good performance of the tracking procedure in **Figure 3A**, where all trajectories except one appear to have been correctly built. The one exception is an insect close to the centre of that figure which appears to have had its trajectory split in three parts (seen as three different coloured segments), most likely due to occlusion by another insect. **Figure 3B** shows the result of constraining these trajectories to a circular region of interest to remove directional bias (in this case, this happened to solve the aforementioned split trajectory, but only by coincidence—the orange and purple tracks were removed for overlapping the edge of the circle).

### 2.5 Implementation

Our implementation of Camfi and its associated tools is written in Python 3.9 (Python Software Foundation, https://www.python.org/). The latest version of Camfi relies on (in alphabetical order): bces 1.0. 3 (28), exif 1.3.1 (29), imageio 2.9.0 (30), Jupyter 1.0.0 (31), Matplotlib 3.4.2 (32), NumPy 1.21.1 (33), Pandas 1.3.0 (34), Pillow 8.3.1 (35), pydantic 1.8.2 (36), Scikit-image 0.18.2 (37), Scikit-learn 0.24.2 (38), SciPy 1.7.0 (39), Shapely 1.7.1 (40), skyfield 1.39 (41), Statsmodels 0.12.2 (42), strictyaml 1.4.4, PyTorch 1.9.0 (43), TorchVision 0.10.0 (44), and tqdm 4.61.2 (45).

Camfi is open source and available under the MIT license. The full source code for the latest version of Camfi and all analyses presented in this paper are provided at https://github.com/J-Wall/camfi. The documentation for Camfi is provided at https://camfi.readthedocs.io/. Camfi is under active development and we expect new features and new trained models to be added as new versions of camfi are released from time to time. All analyses presented in this paper were done using Camfi 2.1.4, which is permanently available from the Zenodo repository https://doi.org/10.5281/zenodo.5242596 (46).

## 3 Results

**Figure 4:**
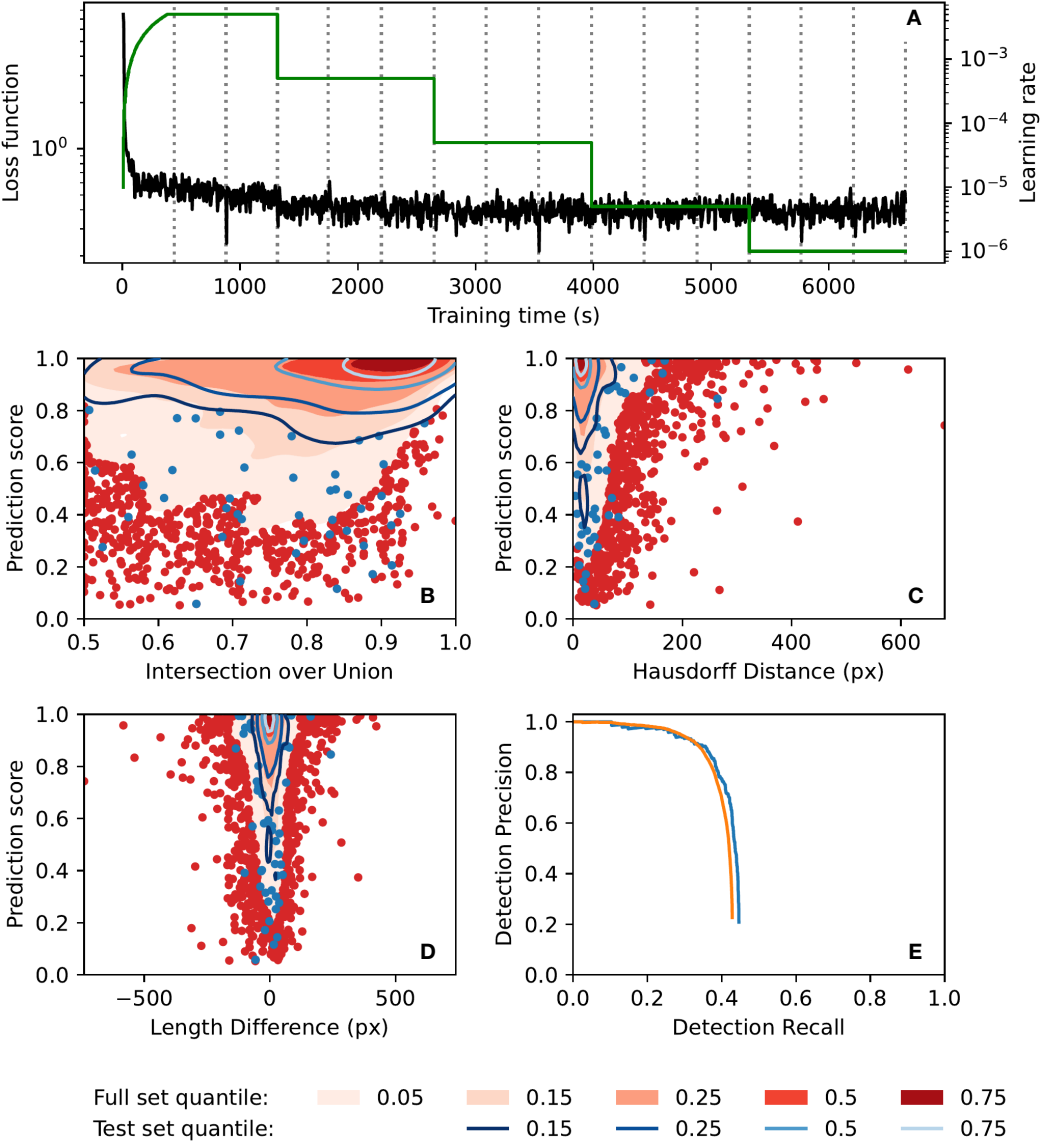
Automatic annotation evaluation plots. **(A)** Automatic annotation model training learning rate schedule (*green*) and loss function (*black*) over the course of training. Epochs (complete training data traversal) are shown with dotted vertical lines. **(B–E)**. Similar performance was seen for both the full 42420-image set (red) and the test 250-image set (*blue*). Gaussian kernel density estimate contour plots of prediction score vs. **(B)** bounding box intersection over union, **(C)** polyline Hausdorff distance, and **(D)** polyline length difference, for both image sets. Contours are coloured according to density quantile (key at bottom of figure). In each plot, data which lie outside of the lowest density quantile contour are displayed as points. **(E)** Motion blur detection precision-recall curve, generated by varying prediction score threshold. The precision-recall curve for the set of images which had at least one manual annotation is shown in *orange*.

Automatic annotation performance was evaluated using a test set of 250 images, as well as the full set of 42420 images. Contour plots of evaluation metrics for both sets are presented in **Figure 4**. The metrics presented are prediction score vs. intersection over union, polyline Hausdorff distance, and polyline length difference (**Figures 4B–D**, respectively; see **Supplementary S3** for definitions of these terms). These plots show similar performance on both the full image set (42420 images) and the test set (250 images), indicating that the model did not suffer from overfitting. Furthermore, they show that prediction scores for matched annotations (automatic annotations which were successfully matched to annotations in the manual ground-truth dataset) tended to be quite high, as did the intersection over union of those annotations, while both polyline Hausdorff distance and polyline length difference clustered relatively close to zero. The precision-recall curves of the automatic annotator (**Figure 4E**) show similar performance between the image sets and show a drop in precision for recall values above 0.6. Training was completed in less than 2 h (**Figure 4A**) on a machine with two 8-core Intel Xeon E5-2660 CPUs running at 2.2GHz and a Nvidia T4 GPU, and inference took on average 1.15 s per image on a laptop with a 6-core Intel Xeon E-2276M CPU running at 2.8GHz and a Nvidia Quadro T2000 GPU.

## 4 Discussion

This paper demonstrates the utility of inexpensive wildlife cameras for the long-term population monitoring and observation of flying behaviour in flying insects. We do not expect this method to completely replace other approaches for monitoring insects, such as trapping, which enables precise measurement of biodiversity and positive identification of species. Likewise, it will not completely replace other remote sensing approaches, such as radar and lidar, which facilitate detecting targets at long distances. However, it is clear that this method has significant potential for complementing these other approaches, and in certain circumstances, replacing them. For instance, in comparison to these other approaches, this method is particularly suited to monitoring assemblages of known species in remote areas, especially when it is known that the target insects are low-flying. An advantage of the presented method over trapping is that much greater temporal resolution is gained, and the sampling rate can easily be adjusted depending on the research question, simply by changing the settings on the cameras. This is in contrast to trapping studies, where typically only one measurement of abundance can be recorded per visit to the trap by the researcher. This provides an opportunity to use the present method to answer a variety of ethological research questions which may not be approachable with previous methods.

This paper has presented a method for monitoring nocturnal flying insects, however there is no reason it could not be used for diurnal species as well, provided care is taken with regard to the placement of cameras. Namely, it would be important to have a relatively uniform background (such as the sky) in order to be able to see insects in the images during the day. In this case, the infra-red flash of the cameras would not be used and the insects would appear as dark objects on a light background. During the day, the exposure time of the cameras is much shorter than at night, so it would be impossible to use this method to measure wingbeat frequencies of day-flying insects. However, in some cases it may be possible to identify day-flying insects in the images directly. It may also be possible to recreate the type of images seen during the night in any lighting conditions by retrofitting the cameras with long-pass infra-red filters, neutral density filters, or a combination of both.

A key advantage of the present method over other approaches is that it can be readily scaled to large monitoring studies or programs, thanks to the low cost of implementation and the inclusion of the tool for automatic annotation of flying insect motion blurs. It is expected that studies implementing this method for target species which substantially differ in appearance from Bogong moths when in flight (and where the use of automatic annotation is desired) may have to re-train the Mask R-CNN instance segmentation model. We believe that the tools we have implemented make that process highly accessible.

## Data availability statement

Publicly available datasets were analyzed in this study. This data can be found here: https://doi.org/10.5281/zenodo.5194496.

## Ethics statement

Ethical review and approval was not required for the study on animals in accordance with the local legislation and institutional requirements.

## Author contributions

JW, JZ, and EW conceived the ideas. JW, TR, and DD conducted the fieldwork. TR and JW performed the manual annotations. JW wrote the software. JW, BB, and DD performed the analyses. JW wrote the first draft of the manuscript with input from EW. and all authors edited the manuscript until completion. All authors contributed to the article and approved the submitted version.
