# Supplementary material for: Camera-based automated monitoring of flying insects (Camfi). I. Field and computational methods
Source: Front Insect Sci. 2023 Sep 13;3:1240400. doi: 10.3389/finsc.2023.1240400 (PMC10926415; doi:10.3389/finsc.2023.1240400)
Supplement: Supplementary file 1 [file DataSheet_1.pdf]

Supplementary Material

Camera-based automated monitoring of flying insects (Camfi). I. Field and computational methods

Wallace, J.R.A.*, Reber, T., Dreyer, D., Beaton, B., Zeil, J., Warrant, E.J. *

*** Correspondence:** Jesse Wallace: jesse.wallace@csiro.au, Eric Warrant: eric.warrant@biol.lu.se

S1. Automated annotation of still images

S1.1 Training

To simplify training of the model to target other species, we have implemented a tool which automates the training process, and this is described below. This tool is packaged with Camfi and is used by running `camfi train` from the command-line. We have also included the model which we trained with Camfi, so for species whose appearance is like that of Bogong moths while in flight, re-training the model may not be necessary.

We adopted the Mask R-CNN model architecture (He et al., 2017) with a Feature Pyramid Network (FPN) backbone (Lin et al., 2017). In the Mask R-CNN framework, the “head” of the model, which is the part of the model which generates the output, can include a variety of branches (corresponding to each type of output). We included the “class”, “bounding box”, and “instance segmentation” branches provided by the Mask R-CNN framework. We assigned two target classes; one for moths and another for background. We initialised the FPN backbone with a model which was pre-trained on the Common Objects in Context (COCO) object segmentation dataset (Lin et al., 2014) and employed the method of transfer learning to train the head and fine-tune the model. The data used for training were the set of images of flying moths we had previously annotated manually which contained at least one moth, as well as the corresponding manual annotations. The training data were augmented by creating new images by horizontal reflection of random individual images. In each iteration of training, a batch of five images were used. The model was trained for 15 epochs (full traversals of training data).

The manual annotations we used were polylines, points, and circles. However, Mask R-CNN operates on bounding boxes and segmentation masks, so some pre-processing of the annotations is required. These pre-processing steps are performed by our software directly on the output of the manual annotation process in VIA. For training the model, bounding boxes and segmentation masks are calculated on-the-fly from the coordinates of the manually annotated polylines, circles, and points. The bounding boxes are simply taken as the smallest bounding box of all coordinates in an annotation, plus a constant margin of ten pixels. The masks are produced by initialising a mask array with zeros, then setting the coordinates of the annotation in the mask array to one, followed by a morphological dilation of five pixels. For polyline annotations, all points along each of the line segments are set to one, whereas for point or circle annotations, just the pixel at the centre of the annotation is set.

We have made our annotation model available as part of the Camfi software and is the default model used by `camfi annotate`. We expect it to work out-of-the box for target species which are similar in appearance to the Bogong moth.

S1.2 Inference

Automation of the inference steps described in this section is implemented in the `camfi annotate` command-line tool, included with Camfi. In inference mode, the Mask R-CNN model outputs candidate annotations for a given input image as a set of bounding boxes, class labels, segmentation masks (with a score from 0 to 1 for each pixel belonging to a particular object instance), and prediction scores (also from 0 to 1). Non-maximum suppression on candidate annotations is performed by calculating the weighted intersection over minimum (IoM) of segmentation masks of each pair of annotations in an image (the definition of IoM is provided below in section S3). For annotation pairs which have an IoM above 0.4, the annotation with the lower prediction score is removed. This has the effect of removing annotations which are too similar to each other and are likely to relate to the same target. We also rejected candidate annotations with prediction scores below a given threshold. For each of the remaining candidate annotations, we fit a polyline annotation using the method described below.

To fit a polyline to a candidate annotation predicted by the Mask R-CNN model, we first perform a second-order polynomial regression on the coordinates of each pixel within the bounding box, with weights taken from the segmentation mask. If the bounding box is taller than it is wide, we take the row (y) coordinates of the pixels to be the independent variable for the regression, rather than the default column (x) coordinates. We then set the endpoints of the motion blur as the two points on the regression curve which lie within the bounding box, and which have an independent variable coordinate ten pixels away from the edges of the bounding box. The rationale for setting these points as the end points is that the model was trained to produce bounding boxes with a ten-pixel margin from the manual polyline annotations (see above). The curve is then approximated by a piecewise linear function (a polyline) by taking evenly spaced breakpoints along the curve such that change in angle between two adjoining line segments is no greater than approximately 15°.

Finally, a check is performed on the polyline annotation to determine if the motion blur it represents is completely contained within the image. If it is not, it is converted to a circle annotation by calculating the smallest enclosing circle of all the points in the polyline annotation using Welzl's algorithm (Welzl, 1991). The check is performed by measuring how close the annotation is to the edge of the image. If the annotation goes within 20 pixels of the edge of the image then the motion blur is considered to not be completely contained within the image, and therefore the polyline annotation is converted to a circle annotation.

The automatically produced annotations are saved to a VIA project file, and tagged with their prediction score, enabling further downstream filtering or annotation visualisation and diagnostics, as well as editing by a human if desired. We ran automatic annotation on the entire image set on a laptop with a Nvidia Quadro T2000 GPU. Using the GPU for inference is preferred, since it is much faster than using the CPU. However, in some cases, images which had a lot of moths in them could not be processed on the GPU due to memory constraints. To solve this problem, `camfi annotate` provides an option to run inference in a hybrid mode, which falls back to the CPU for images which fail on the GPU.

S1.3 Validation

This section introduces a number of terms which may be unfamiliar to the reader. Definitions of the following terms are provided in section S3: intersection over union, Hausdorff distance, signed length difference, precision-recall curve. As mentioned in the main text, we kept 250 randomly-selected annotated images as a test set during model training. We ran inference and validation on the full set of images, and on the test set in isolation. For both sets, we matched automatic annotations to the ground-

truth manual annotations using a bounding-box intersection over union (IoU) threshold of 0.5. For each pair (automatic and ground-truth) of matched annotations we calculated IoU, and if both annotations were polyline annotations, we also calculated the Hausdorff distance and the signed length difference between the two annotations. Gaussian kernel density estimates of prediction score versus each of these metrics were plotted for diagnostic purposes and presented in main paper Fig. 4. We also plotted the precision-recall curve for both image sets.

S2. Wingbeat frequency measurement

For observations of moths whose motion blur was entirely captured within the frame of a camera, we use a polyline annotation, which follows the path of the motion blur. This annotation can be obtained either manually or automatically, by the procedures described above. Since the moth is moving while beating its wings, we are able to observe the moth's wingbeat (see main paper Fig. 1b). Incorporating information about the exposure time and rolling shutter rate of the camera, we are able to make a measurement of the moth's wingbeat frequency in hertz. We have implemented the procedure for making this measurement as part of Camfi, in the sub-command called `camfi extract-wingbeats`. The procedure takes images from wildlife cameras (like those shown in main paper Fig. 1b,c) and a VIA project file containing polyline annotations of flying insect motion blurs as input, and outputs estimates of wingbeat frequencies and other related measurements. A description of the procedure for a given motion blur annotation follows.

First, a region of interest image of the motion blur is extracted from the photograph, which contains a straightened copy of the motion blur only (see Fig. S1a). The precise method for generating this region of interest image is not important, provided it does not scale the motion blur, particularly in the direction of the motion blur. Our implementation simply concatenates the rotated and cropped image rectangles, which are centred on each segment of the polylines, with length equal to the respective segment, and with an arbitrary fixed width. We used the default value of 100 pixels.

The pixel-period of the wingbeat, which we denote P, is determined from the region of interest image by finding peaks in the autocorrelation of the image along the axis of the motion blur (see Fig. S1b). The signal-to-noise ratio (SNR) of each peak is estimated by taking the Z-score of the correlation at the peak, if drawn from a normal distribution with mean and variance equal to those of the correlation values within the regions defined by the intervals $\left(\frac{1}{4}P^*, \frac{3}{4}P^*\right) \cup \left(\frac{5}{4}P^*, \frac{7}{4}P^*\right)$, where P^* is the pixel period corresponding to the given peak. The peak with the highest SNR is selected as corresponding to the wingbeat of the moth and is assigned as P. The total length of the motion blur (in pixels) may then be divided by P to obtain a non-integer wingbeat count for the motion blur. The SNR of the best peak is included in the output of the program, to allow for filtering of wingbeat data points with low SNR. It should be noted that the definition of SNR used here may differ somewhat from other formal definitions. For example, this definition admits negative values for SNR (albeit rarely), in which case the corresponding measurement will surely be filtered out after a SNR threshold is applied.

When running camfi extract-wingbeats, supplementary figures containing the region of interest images and corresponding autocorrelation plots, similar to those presented in Fig. S1 can be optionally generated for every polyline annotation.

To calculate wingbeat frequency F_w, in hertz, we need to know the length of time that the moth was exposed to the camera, which we call Δt. Unfortunately, this is not as simple as taking the exposure

time as reported by the camera, which we call t_e, due to the interaction of the first-order motion of the moth with the rolling shutter of the camera. In particular,

$$\Delta t = t_e \pm \frac{|r_1 - r_0|}{R} \quad , \qquad \text{(Equation 1)}$$

where r_0 and r_1 are the row indices (counting rows from the top of the image) of the two respective ends of the polyline annotation, and R is the rolling shutter line rate, which was measured to be 9.05×10^4 lines/second for the cameras we used (for a method of measuring R, see the Camfi documentation at https://camfi.readthedocs.io/). The "±" reflects the fact that it is impossible to tell in which direction the moth is flying from the images alone, leading us to two possible measurements of moth exposure time, corresponding to the moth flying down or up within the image plane of the camera, respectively. Under certain circumstances, this ambiguity can be resolved by observing that $\Delta t \geq 0$, i.e. insects cannot fly backwards through time. Intuitively, we may then attempt to calculate F_w by dividing the wingbeat count by Δt (these preliminary estimates of wingbeat frequency are included in the output of `camfi extract-wingbeats`). However, this would require the assumption that the moth has a body length of zero, since the length of the motion blur, which we denote as L, is the sum of the first order motion of the moth during the exposure and the moth's body length, projected onto the plane of the camera. Clearly, this assumption may be violated, as the insects have a non-zero body length in the images. We denote the body length of the moth projected onto the plane of the camera by the random variable L_b.

The statistical procedure for estimating the mean and standard deviation of observed moth wingbeat frequency, which accounts for both the time ambiguity and the non-zero body lengths of the moths, is as follows. We begin with the following model, which relates Fw to Lb and various measured variables.

$$L_i = F_{w_i} P_i \Delta t_i + L_{b_i} \quad , \qquad \text{(Equation 2)}$$

where i is the index of the observation. We proceed by performing a linear regression of L on $P\Delta t$ (setting $P\Delta t$ as the independent variable) using the BCES method (Akritas and Bershady, 1996) to obtain unbiased estimators of $\overline{F_w}$ and $\overline{L_b}$, as well as their respective variances, $\sigma^2_{F_w}$ and $\sigma^2_{L_b}$. Values for Δt_i are taken as the midpoints of the pairs calculated in Eq. 2, with error terms equal to $\frac{\left|r_{1_i} - r_{0_i}\right|}{R}$. Values for L_i are assumed to have no measurement error. Where multiple species with different characteristic wingbeat frequencies are observed, an expectation-maximisation (EM) algorithm may be applied to classify measurements into groups which may then be analysed separately. We may then test the zero body-length assumption, namely $\overline{l_b} = 0$, by calculating its t-statistic.

S3. Definitions of automatic annotation validation metrics

S3.1 Weighted Intersection over Minimum (IoM)

It is common for automatic image annotation procedures to produce multiple candidate annotations for a single object of interest (in our case, motion blurs of flying insects). It is therefore necessary to perform non-maximum suppression on the automatically generated candidate annotations (where only the best candidate annotation for each object is kept).

In order to perform non-maximum suppression on candidate annotations, we need a way of matching annotations which refer to the same object. This is typically done by defining some measure of similarity between two annotations, and then applying this measure to each pair of annotations within an image. Then, by setting an appropriate threshold, the program can decide which pairs of annotations require non-maximum suppression to be applied.

In the case of our method, it is common for the automatic annotation procedure to produce multiple annotations of different sizes for each motion blur. This is likely due to the fact that the motion blurs themselves can vary greatly in length and in number of wingbeats, which causes some level of confusion for the automatic annotation model. Therefore, we need to use a similarity measure which is invariant to the size of annotations and produces a high similarity for annotations which are (roughly) contained within each other. This motivates our definition of similarity of candidate annotations for the purposes of non-maximum suppression. Namely, weighted intersection over minimum (IoM).

Suppose we have two sets $A = \{a_0, a_1, \dots, a_n\}$ and $B = \{b_0, b_1, \dots, b_n\}$ with corresponding weights $X = \{x(a_0), x(a_1), \dots, x(a_n)\}$ and $Y = \{y(b_0), y(b_1), \dots, y(b_n)\}$. We define the intersection over minimum of the two sets as

$$IoM(A,B) = \frac{\sum_{z \in A \cap B} \min\big(x(z), y(z)\big)}{\min(\sum X, \sum Y)}$$

In Camfi, we apply this definition by setting the weights X and Y as the segmentation mask values from two candidate annotations respectively. In this case, $A = B$ are the coordinates of every pixel in the image.

S3.2 Bounding-box Intersection over Union (IoU)

To validate the quality of an automatic annotation system, we would like to compare the annotations produced by the system to annotations produced by a human. To do this, we need to have a way of matching pairs of annotations. This can be done by measuring the similarity between two annotations, and if they are similar enough, matching them.

The bounding-box intersection over union (IoU) is a commonly used similarity measure for object detection on images. It is defined as per its name. That is, we find the bounding box of two annotations, then calculate the ratio of the intersection of the two boxes with the union of the two boxes.

S3.3 Hausdorff distance

Our method for measuring wingbeat frequencies depends on accurate annotations of flying insect motion blurs, so it is important to know the accuracy of the annotations produced by our method for automatic annotation.

Suppose we have an automatically generated polyline annotation, and a corresponding polyline annotation made by a human which we would like to validate the automatic annotation against. We would like to know how accurately the automatic annotation recreates the human annotation. We proceed by calculating the Hausdorff distance between the two annotations. First, we define two sets A and B which contain all the points on the respective polyline from each of the two annotations.

The Hausdorff distance $d_H(A, B)$ is defined as

$$d_H(A,B) = \max\left\{\sup_{a\in A}\inf_{b\in B} d(a,b), \sup_{b\in B}\inf_{a\in A} d(a,b)\right\} \quad ,$$

where $d(a,b)$ is the Euclidean distance between points a and b. In other words, the Hausdorff distance is the maximum distance between a point in one of the sets, to the closest point in the other. For the purpose of validating automatic annotations, we see that smaller Hausdorff distances between the automatic and manual annotations are better than larger ones.

S3.4 Signed Length Difference

Another way to assess the accuracy of the automatic polyline annotations against the manually produced annotations is signed length difference ΔL . This is motivated by the fact that our method for calculating wingbeat frequency is fairly sensitive to the length of the polyline annotation. Suppose we have an automatically generated polyline annotation with length L_A and a corresponding ground-truth manual annotation with length L_G. Then the signed length difference is defined as $\Delta L = L_A - L_G$. The closer the signed length difference is to zero, the better.

S3.5 Precision-Recall curve

With regard to object detection, precision is the proportion of detections which correspond to annotations present in the ground-truth dataset. Recall is the proportion of objects in the ground-truth dataset which are detected by the automatic annotation system. In our case, the ground-truth dataset is the set of manual annotations. We match automatic annotations with ground-truth annotations if they have an IoU greater than 0.5.

Each candidate annotation is given a confidence score between 0.0 and 1.0 by the annotation model. This score can be used to filter the candidate annotations (e.g. by removing all annotations with a score less than 0.9). By varying the score threshold, we obtain different precision and recall values for the system.

A precision-recall curve is the curve drawn on a plot of precision vs. recall by varying the score threshold. The closer the curve goes towards the point (1, 1), the better.

S4. References

Akritas, M.G. and Bershady, M.A. (1996). Linear regression for astronomical data with measurement errors and intrinsic scatter. *Astrophysical J*. 470, 706–714. https://doi.org/10.1086/177901

He, K., Gkioxari, G., Dollár, P., Girshick, R., 2017. Mask R-CNN, in: Proc. *IEEE Int. Conf. Computer Vision*. pp. 2961–2969. https://doi.org/10.1109/iccv.2017.322

Lin, T.-Y., Dollár, P., Girshick, R., He, K., Hariharan, B., Belongie, S., 2017. Feature pyramid networks for object detection, in: *Proc IEEE Conf Computer Vision and Pattern Recognition*. pp. 2117–2125. https://doi.org/10.1109/cvpr.2017.106

Lin, T.-Y., Maire, M., Belongie, S., Hays, J., Perona, P., Ramanan, D., Dollár, P., Zitnick, C.L., 2014. Microsoft COCO: Common objects in context, in: *Euro. Conf. Computer Vision*. Springer, pp. 740–755. https://doi.org/10.1007/978-3-319-10602-1_48

Welzl, E., 1991. Smallest enclosing disks (balls and ellipsoids), in: *New Results and New Trends in Computer Science*. Springer, pp. 359–370. https://doi.org/10.1007/BFb0038202

S5. Supplementary Figures

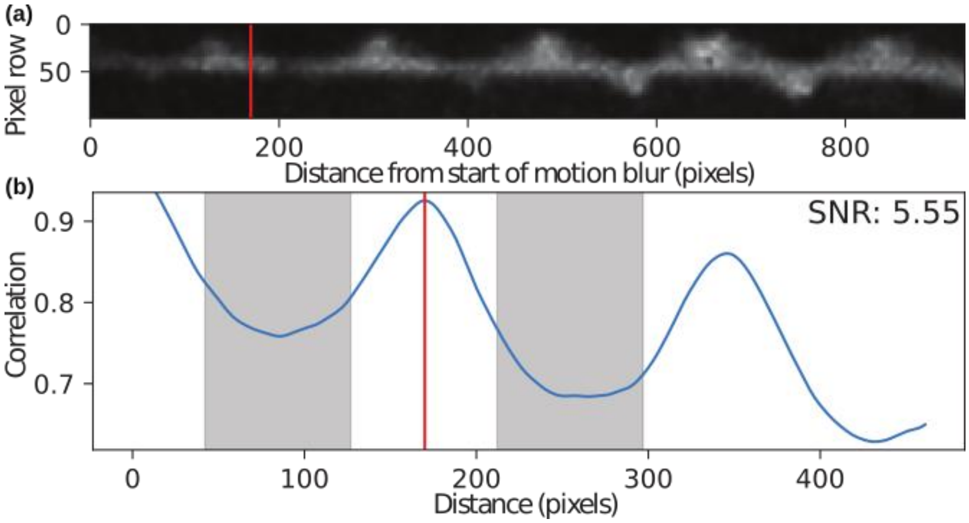

Supplementary Figure S1. (a) Straightened and cropped "region-of-interest image" of moth motion blur, taken from image shown in main paper Fig. 1b. Red vertical line shows periodicity along the axis of the motion blur as calculated by our algorithm. **(b)** Autocorrelation of region-of-interest image (shown in **a**) along the axis of the motion blur. Red line shows peak periodicity as calculated by our algorithm. Signal-to-noise ratio (SNR) is calculated as the Z-score of the correlation at the peak, if drawn from a normal distribution with mean and variance equal to those of the correlation values within the shaded regions, defined by the intervals $\left(\frac{1}{4}P^*, \frac{3}{4}P^*\right) \cup \left(\frac{5}{4}P^*, \frac{7}{4}P^*\right)$, where P^* is the pixel period.
